# Innate Immunity: A Balance between Disease and Adaption to Stress

**DOI:** 10.3390/biom12050737

**Published:** 2022-05-23

**Authors:** Irene Faenza, William L. Blalock

**Affiliations:** 1 Dipartimento di Scienze Biomediche e Neuromotorie, Università di Bologna, 40126 Bologna, Italy; 2 “Liugi Luca Cavalli-Sforza” Istituto di Genetica Molecolare, Consiglio Nazionale delle Ricerche, 40136 Bologna, Italy; 3 IRCCS Istituto Ortopedico Rizzoli, 40136 Bologna, Italy

**Keywords:** inflammation, innate immunity, cancer, autoinflammation, neuro-muscular degeneration, Fanconi anemia, adaption

## Abstract

Since first being documented in ancient times, the relation of inflammation with injury and disease has evolved in complexity and causality. Early observations supported a cause (injury) and effect (inflammation) relationship, but the number of pathologies linked to chronic inflammation suggests that inflammation itself acts as a potent promoter of injury and disease. Additionally, results from studies over the last 25 years point to chronic inflammation and innate immune signaling as a critical link between stress (exogenous and endogenous) and adaptation. This brief review looks to highlight the role of the innate immune response in disease pathology, and recent findings indicating the innate immune response to chronic stresses as an influence in driving adaptation.

## 1. Introduction

Since first being documented in ancient times by Greek and Egyptian physicians, the relationship of inflammation with injury and disease has evolved in complexity as well as causality. Initial characteristic descriptions by Hippocrates (5th century BC) included swelling or “edema” of the affected tissue. Later, Aulus Celsus (1st century BC/1st century AD) described the four main manifestations of inflammation: pain, edema, warmth, and redness of the interested tissue. The Roman physician/surgeon Galen later added the fifth hallmark of inflammation—loss of function of the affected tissue—a hallmark most closely associated with muscular, bone, or joint manifestations of inflammation visible, at the time, upon examination of the patient [1]. The empirical observations set the framework for detailed scientific studies over the centuries that resulted in the discovery and characterization of the immune system and its role in the response to not only injury, but also disease. These early studies supported a cause-and-effect theory whereby injury or disease gave rise to inflammation. However, is inflammation simply a case of the immune system responding to disease or can inflammation have a causative role in disease? The number of pathologies now linked to chronic inflammation would suggest inflammation may be able to both promote and be the initiating stimulus in multiple pathologies, but to this day the inflammation–disease relationship remains controversial. To clearly understand this relationship, one must first understand the relationship between inflammation, innate and acquired (or cell-based) immunity, DNA repair, and the assortment of mutations and epigenetic modifications accumulated during chronic inflammation.

## 2. Innate Immunity vs. Adaptive Immunity

**Figure 1:**
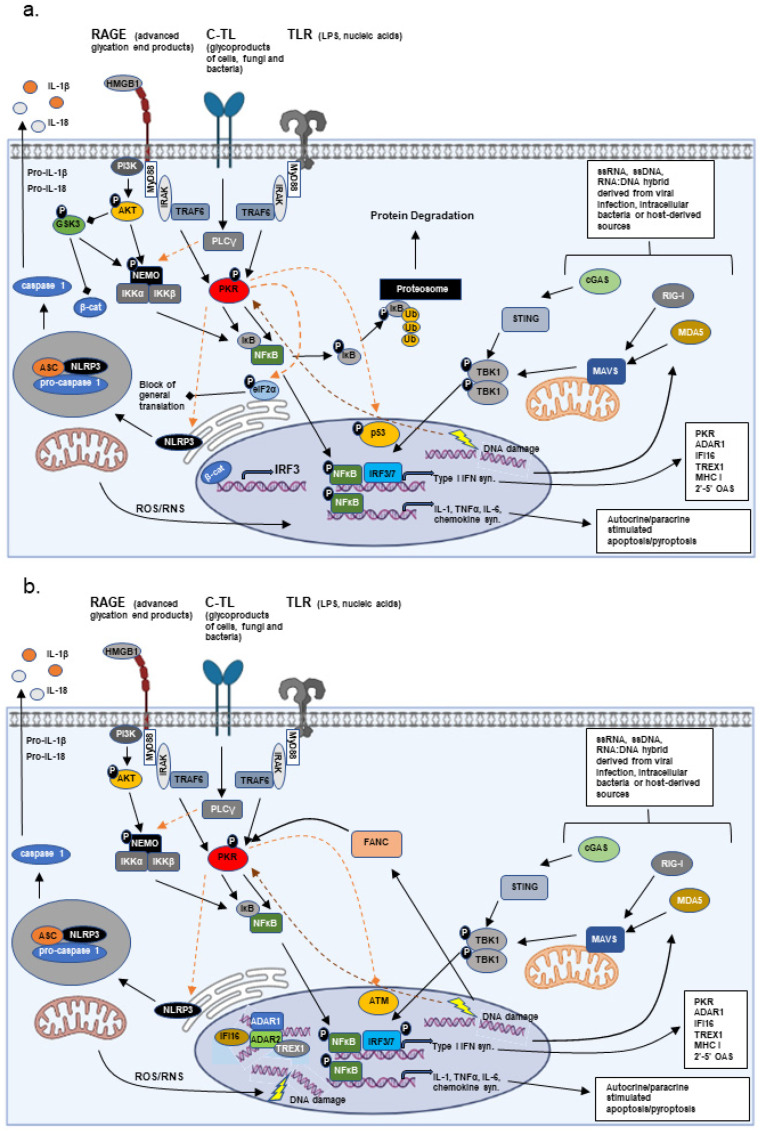
Innate Immune Signaling. The major signal transduction pathways mediated by extracellular and intracellular Pattern Recognition Receptors (PRRs) of both Pathogen-Associated Molecular Patterns (PAMPs) and Damage-Associated Molecular Patterns (DAMPs) are presented. (**a**) Extracellular pathogens, stressors, and products from damaged cells can result in the stimulation of the receptor for advanced glycation end products (RAGE; high mobility group box (HMGB)-1, advanced glycation end products (AGE)), C-type lectin (C-TL; glycoproducts of bacteria, fungi) or Toll-like receptors (TLR; nucleic acids, LPS, glycoproteins), primarily resulting in the activation of nuclear factor-κB (NF-κB) through signaling mediated by AKT and/or PKR. AKT stimulates survival, while PKR phosphorylates eukaryotic initiation factor 2α (eIF2α) and p53, inhibiting general translation and promoting p53 stability, respectively. Additionally, PKR results in both the phosphorylation and degradation of inhibitor κB (IκB) and the phosphorylation of p65 NF-κB, promoting the translocation of p65 NF-κB to the nucleus and the stimulation of NFκB-dependent transcripts, including cytotoxic cytokines and type I interferon. Activation of the intracellualar PRRs (RIG-I, MDA5, or cGAS) promote the phosphorylation and activation of interferon response factors (IRF)-3 and -7. In conjunction with p65 NF-κB, IRF3/7 stimulates the synthesis of type I interferon, which is secreted, thereby having both paracrine and autocrine effects. The associated signaling can promote cell death as well as the recruitment of immune cells to the site of damage/stress. AKT-mediated suppression of GSK3 promotes β-catenin (β-cat) translocation to the nucleus and the synthesis of IRF3. In many autoinflammatory/degenerative diseases, GSK3 remains active and interferon signaling is defective. (**b**) Under chronic stress conditions (environmental factors, DNA repair defects (FANC)), prolonged activation of the PRRs and interferon-inducible kinase PKR leads to phosphorylation and inactivation of the DNA repair kinase, ATM, and an enhancement in the rate of mutagenesis. In addition, a number of interferon-inducible proteins, which act as both epigenetic modifiers and DNA repair proteins, associate with sites of DNA strand breaks, potentially regulating the mutation incorporation rate.

The immune response is broken down into innate immune and acquired immune responses. The innate immune response centers around a cell’s ability to recognize foreign agents and organisms or recognize damage of itself or neighboring cells [2]. Foreign agents or infectious organisms are recognized by Pattern Recognition Receptors (PRRs) that bind Pathogen-Associated Molecular Patterns (PAMPs), while cellular damage is recognized by PRRs that interact with Damage-Associated Molecular Patterns (DAMPs) [2,3]. The PRRs are classified by their cellular localization. PRRs localized to the cellular membrane include the Toll-like receptors (TLRs 1–10 in humans), C-type lectin receptors (CLRs), and the receptor for advanced glycation end-products (RAGE). Each of the ten TLRs are activated by distinct and specific stimuli, ranging from proteins to carbohydrates to nucleic acids [3]. Similarly, the CLRs are stimulated by specific binding of molecules which may or may not be a carbohydrate [3]. In contrast, the RAGE receptor binds a number of alarmins that include: advanced glycation end products (AGEs), high mobility group box (HMGB)-1, and amyloid-β protein [4]. Cell membrane-associated PRRs promote phagocytosis and the activation of nuclear factor (NF)-κB, thus promoting antigen presentation and the synthesis of diverse pro-inflammatory cytokines (interleukin (IL)-1β, tumor necrosis factor (TNF)-α, IL-6, and interferon (IFN)-γ), respectively. These changes contribute to the recruitment and activation of additional immune cells more closely associated with the acquired immune response (Figure 1).

In contrast, PRRs localized to the cytosol include retinoic acid-inducible gene (RIG)-I, melanoma differentiated protein (MDA)-5, probable ATP-dependent RNA helicase DHX58 (LGP2), cyclic GMP-AMP synthase (cGAS), protein kinase dependent on double-stranded (ds) RNA (PKR), the nucleotide-binding oligomerization domain proteins (NOD1 and NOD2), and the NOD-like receptor proteins (ex. nucleotide-binding oligomerization domain, leucine rich repeat, and pyrin domain containing (NLRP)). RIG-I, MDA5, and cGAS recognize distinct nucleotide molecules leading to the activation and nuclear localization of the interferon response factors (IRF)-3 and -7, thus promoting the synthesis of type I interferons [5,6]. In contrast, the NODs and NOD-like receptors bind a variety of microbial or cellular molecules, leading most notably to NF-κB activation, caspase 1 activation, and synthesis of IL-1 [7]. Interestingly, PKR is a PRR which carries-out a number of diverse functions [8]. Similar to RIG-I and MDA5, PKR binds dsRNA. Binding of dsRNA or association with the PKR activator protein PACT leads to enzymatic activation of PKR ([9,10]). Active PKR results in the phosphorylation and nuclear translocation of p65 NF-κB, where it synergizes with IRF-3 and -7 to induce type I IFN synthesis [11]. In addition, active PKR phosphorylates eukaryotic translation initiation factor (eIF)-2α, promoting inhibition of general translation and thereby blocking the synthesis of most proteins [12]. PKR activation also stimulates activation of the NLRP3 inflammasome, promoting the synthesis of IL-1β [13]. Several of these cytosolic PRRs are known to be required downstream for the proper signaling of certain membrane-associated PRRs, such as TLRs, and may or may not involve nucleotide binding [14]. Both PKR and RIG-I have been shown to be activated by RAX/PACT by varying stresses in a dsRNA-independent manner as well, and loss of RAX/PACT severely hampers PKR and RIG-I activation, even under conditions of viral infection where dsRNAs are plentiful [9,10,15,16] (Figure 1).

All cells and tissues are capable of initiating an innate immune response in one form or another. In the short-term, these responses have the purpose to limit the spread of an infectious agent or limit the extent of cell and tissue damage, give time for cellular repair, as well as certify that any unrepaired damage cannot be propagated further. These responses also result in the increased synthesis and expression of chemotactic factors that recruit additional circulating cells (macrophages, neutrophils, and natural killer (NK) cells) to the site of inflammation [17]. While the affected cells and tissues can express inflammatory cytokines and present antigens, certain recruited cells, like macrophages, are professional antigen-presenting cells (APCs) and producers of both inflammatory and anti-inflammatory cytokines that are instrumental in regulating the acquired immune response (adaptive immunity) [18]. Even though certain cell types, such as macrophages and natural killer cells, serve an important role in the innate immune response, innate immunity is generally considered to be non-cell mediated, as it does not require the involvement of specialized immune cells (T- and B-lymphocytes). 

The adaptive immune response mainly centers on the involvement of both T and B lymphocytes. Antigen-presenting cells present surface-bound antigens to both B and T cells in the context of the antigen receptor and the presence of a co-stimulatory receptor [18]. It is often the context of this presentation that dictates the ensuing response. All cells express major histocompatibility complex class I (MHC I) and are capable of presenting intercellular antigens and recruiting CD8^+^ cytotoxic T lymphocyte (CTL) involvement. In contrast to MHC I, not all cell types express MHC II. Previously, it was assumed that MHC II expression was limited to macrophages, dendritic cells, and B cells, but in addition to these, most myeloid lineage cells (neutrophils, basophiles, mast cells, and eosinophiles) and endothelial cells have been reported to express MHC II [18,19]. Presentation of antigen in the context of MHC II stimulates CD4^+^ T lymphocytes (T helper cells), IL-2 expression, and the expansion of CD4^+^ T helpers. Again, depending on the context, these activated T helper cells can either favor the activation of macrophages and the expansion of CTLs, stimulate B cell activation, the transition of activated B cells to antibody-secreting plasma cells and memory B cells, or, in the case of regulatory T cells (Tregs), suppress effector T cell (Th1 and Th2) activity [20]. 

In response to stress-mediated damage, restoration of homeostasis in the host organism requires concerted interaction between both branches of the immune system. With the exception of circulating antibody or direct engagement of lymphocyte populations, most immune responses to stress initiate through the innate branch of the immune system. DAMPs and PAMPs either stimulate PRRs or are recognized and bound by specialized receptors then directly phagocytized by circulating macrophages, dendritic cells, and neutrophils [2,3,21]. Components of the complement system can influence this early step in the immune response [21]. Stimulation of the PRRs produces a variety of effects due both to the intracellular signaling they stimulate, as well as the synthesis of cytokines and chemokines, which have autocrine and paracrine effects. The type I interferons (IFNα/β) stimulate, in affected and neighboring cells, some of the same intracellular signaling pathways aimed at limiting damage and energy usage as those directly activated by the PRRs [22]. Additional cytokines such as IL-1, IL-6, and TNFα promote diverse inflammatory aspects, which often include enhance generation of reactive oxygen species (ROS), through a variety of mechanisms [23]. Macrophages and neutrophils recruited to the site of inflammation can also be a significant source of ROS. The generated ROS can further stimulate immune cell recruitment and activation, promote the induction of several inflammatory cytokines, or damage macromolecules such as DNA and protein (both pathogen and host) [23]. Many of these cytokines also result in the synthesis of both pro- and anti-apoptotic factors through the activation of transcription factors, including NF-κB and MYC [24,25,26]. The secreted cytokines and chemokines in turn promote the recruitment of immune cells (macrophages, dendritic cells, NK cells, B- and T-lymphocytes) to the site of stress and damage [27]. Type I interferons have additional effects in that they stimulate the enhanced synthesis of MHC I molecules and several anti-inflammatory cytokines, like IL-10, which are key in resolving the inflamed state [22,28].

Enhanced expression of MHC I on the cell surface aids natural killer (NK) cells in distinguishing self from non-self, as well as augments the expression of endogenous/intracellular antigens to the relevant pool of CTLs, by the process of cross-presentation [29]. However, for most cells, the capability to stimulate a full CTL response is limited and requires CD4^+^ T helper 1 (Th1) cells, dendritic cells, or inflammatory (M1) macrophages to supply the co-stimulation (CD40:CD40L and CD28:B7) and additional cytokines, such as IL-2, IL-12, and IFNγ, that are necessary [29,30]. In contrast, the presence of CD4^+^ T helper 2 (Th2) cells, T regulator (Treg) cells, or suppressor macrophages (M2), which interact with CTLs but express a differing cytokine profile (IL-4, IL-10, PD-1), can suppress CTL activation, and are considered key factors in tumor development and progression [30,31]. While many of the cytokines produced by these immune cells are specific regulators of other immune cells, a significant set, including inflammatory cytokines TNFα, IL-6, IL-1, and anti-inflammatory cytokines IL-4 and IL-10, can affect the innate immune response of most cell types and tissues.

Macrophages, dendritic cells, and neutrophils recruited to the site of inflammation can exert differing effects. These cells have the ability not only to present phagocytized or endogenous/intracellular antigens via MHC I, but they also express MHC II and have the ability to present antigens via this complex as well, allowing for the stimulation of T helper cells and the polarization of these to a number of T helper phenotypes (Th1, Th2, Th17, Treg, etc.) based on the mode of antigen presentation and the cytokines present [32]. Likewise, B-lymphocytes carry out a similar antigen-presenting role, but with much more specific antigen recognition. The B-cell antigen receptor (or surface antibody) binds specifically to antigen. The receptor:antigen complex is internalized and the processed antigen presented in complex with MHC II. The ensuing interaction of the B-cell with CD4^+^ T helper cells leads to the activation of the B-cell and the polarization of the T helpers to primarily a Th2 response [32,33]. B-cell activation in conjunction to the secondary process of antibody affinity maturation generally leads to the production of highly specific IgG molecules [34]. Like the polarization of macrophages and T-cells, the cytokines and secondary messengers present can influence these processes, dictating the prevalence of certain B-cell clones and the antibodies they produce. Thus, these cytokines may influence the generation of autoantibodies observed in several autoimmune/inflammatory/degenerative diseases [35]. In addition, a relatively new set of T helper cells has gained increased attention as regards inflammation. T helper cells expressing IL-17, or Th17 cells, are a subset of pro-inflammatory T cells that primarily stimulate neutrophil production and recruitment and the innate immune/inflammatory branch of the immune system [31,36]. As these cells are of CD4^+^ lineage, they are activated in similar ways to Th1 and Th2 T helper cells, but they differ in the cytokine profile they synthesize. Similar to Th1 and Th2, there is also a set of regulatory or suppressor T-cells specific to Th17 cells (Treg17 cells) that result in suppression of the Th17 response [36,37]. The major role of the diverse T helper populations is to coordinate the acquired immune response, either by promoting a cell-based response (CTLs, macrophages, NK cell, neutrophils, etc.), a humoral response (B-cell/antibody), both or neither (suppression). While innate immunity has a leading role in establishing and maintaining the inflammatory environment, it is primarily alterations in acquired immunity, in conjunction to any genomic/epigenetic alterations in the affected tissue, which lead to auto-inflammation or dictate the balance between degenerative disease versus proliferative disease in chronic inflammatory pathologies.

## 3. Chronic Inflammation and Disease

Chronic inflammation is an integral part of multiple pathologies, including degenerative diseases (Alzheimer’s, Parkinson’s, inclusion body myositis), metabolic diseases (diabetes mellitus), autoimmune diseases (multiple sclerosis, scleroderma), and cancer [38]. Chronic inflammation occurs when either the stressing agent or conditions causing stress cannot be resolved, or alterations in the immune response hamper resolution of inflammation, which can lead to off-target damage. It is not surprising that many chronic inflammatory diseases are associated with aging. This is likely the result of three factors: (i) aging tissues tend to be more fragile and prone to damage following stress [39,40,41]; (ii) aging organisms have a reduced stem cell capacity for tissue repair; and (iii) the aging immune system is more prone to errors [42]. Thus, in aging tissue there is an increased propensity for tissue injury and a reduced capacity to repair the injury, compounded by a deficiency in the immune response, thus leading to drawn-out inflammatory responses that can further damage tissue. However, chronic inflammation also has a major role in a number of chronic non-age-related pathologies.

Chronic non-age-related inflammatory pathologies can be sub-divided based on whether (1) an underlying genetic alteration stimulates an innate immune response, (2) the genetic alteration directly affects a component of the immune system, or (3) environmental/behavioral factors (pathogens, toxins) exert a constant stress on the organism. Any one of these would be expected to promote disease. Additionally, there are likely an unknown number of non-pathogenic genetic mutations that in conjunction with the wrong environmental/behavioral factors constitute a predisposing event for disease. While there are too many instances of non-age-related inflammatory pathologies to list here, the following present examples of each type based on their origin. 

### 3.1. Fanconi Anemia (A Genetic Alteration Stimulating an Innate Immune Response)

DNA damage as a result of extrinsic insults (radiation, chemotherapy) or genetic alterations that lead to enhanced accumulation of DNA damage result in the stimulation of DAMPs and PRR proteins [43,44]. In Fanconi anemia (FA) there is significant evidence that the pathology observed is directly related to the innate immune/inflammatory response to enhanced DNA damage [16,45,46,47,48]. Fanconi anemia is a DNA damage repair syndrome that results from mutation in one of 23 genes identified to date [49]. Affected individuals present a heterologous array of symptoms, including short stature, premature aging, congenital defects, anemia, and a propensity for cancer formation. The presence and penetrance of each of these disease characteristics varies depending on the affected FA gene and the mutation present. A hallmark of Fanconi anemia is the enhanced sensitivity of individuals with FA to DNA cross-linking agents (mitomycin C, cisplatin) and pro-inflammatory cytokines (TNFα, IFNγ), making tumors in these patients almost impossible to treat with standard therapy protocols.

Inflammation in response to DNA damage in FA patients likely involves at least two PRRs, resulting in type I IFN synthesis. Under normal conditions, cellular transcription and replication induce cell stress, DNA strand breaks, and the formation of R-loops; thus, DNA damage is a constant challenge for the cell and must be resolved by the DNA repair mechanisms present [50]. In FA, DNA repair is inherently defective, leading to the activation of stress response pathways. Zhang et al. demonstrated that PKR interacted with FANCA, FANCC, and FANCG proteins and that the interaction between PKR and FANCC was enhanced when FANC proteins harboring disease-related mutations were present in primary bone marrow cells or patient-derived lymphoblasts [45]. The interaction of PKR with mutant FANCC or the complete lack of FANCC in cells derived from FANCC-null mice was previously demonstrated by this same group to stimulate PKR activation through a mechanism involving HSP70 [51,52]. In both cases, elevated PKR activation promoted hypersensitivity to DNA cross-linking agents and cytotoxic cytokines, which could be suppressed through overexpression of a dominant-negative form of PKR. These studies were some of the first to demonstrated that PKR activation is a standard response to DNA damage, a view further supported by the finding that PKR is also activated in response to DNA damaging reagents, including bulky adducts, UV- and γ- irradiation [46,53,54]. 

Additionally, enhanced DNA damage and R-loop formation in FA cells produces an accumulation of cytosolic DNA, which stimulates the cGAS-STING pathway, promoting an IFN-like profile [43]. Activation of both PKR and cGAS-associated pathways promote the synthesis of type I IFNs; these, in turn, stimulate the synthesis of interferon regulated genes (see Section 2) which include PKR, adenosine deaminase acting on dsRNA (ADAR)-1, interferon stimulated gene (ISG)-15, RIG-I, and MDA5, to name a few [16,45,46,47]. Several of the induced genes are known to be involved in DNA repair. Among these is ADAR1, which has recently been shown to have roles in R-loop resolution and genome stability [55]. Mutations in ADAR1 are known to cause mild (Dyskeratosis congenita) to severe (Aicardi–Goutieres syndrome) inflammatory pathologies. Similarly, while not interferon-inducible, ADAR2, which often acts in conjunction with ADAR1, was demonstrated to be necessary for DNA double-strand break repair [56].

Disease progression in FA patients often involves anemia progressing to a myelodysplastic syndromes (MDS) stage, followed by full-blown acute myelocytic leukemia (AML). Bone marrow/hematopoietic stem cell (HSC) transplantation has been a great therapeutic advancement for the treatment of the bone marrow and hematologic manifestations of FA, but FA patients also have increased propensity for developing tumors of the oral mucosa [57]. One line of thought for why the oral mucosa is predominantly involved in FA patients is that these locations represent the tissues most exposed to the surrounding environment and are a first line defense of the innate immune response to environmental toxins and stress. Thus, inflammation is believed to be a significant promoting influence in these tumors. Interestingly, while HSC transplantation is a viable treatment for the hematologic manifestations in FA patients, it has been reported to enhance the risk of developing solid tumors at other sites when compared to FA patients not receiving HSC transplantation [57]. One possibility for this paradox could be that, following HSC transplantation (HSCT), these patients now constitute a chimera with normal hematopoietic bone marrow progenitor cells that can give rise to a fully competent immune cell compartment while the remaining cells and tissues are defective, still expressing a mutant FANC protein. This would be predicted to produce an enhanced pro-inflammatory environment with a graft vs. host-like involvement [58]. Additionally, these observations may relate more to the fact that certain FANC mutations have less severe sequelae than others. Individuals who are not in need of HSCT would most likely be those with less severe disease and hence a lower probability of developing oral cancers, while patients in need of HSCT are those harboring FANC mutations that are associated with more severe disease characteristics and conceivably a higher probability of developing oral cancers. Therefore, patients requiring HSCT would have an inherently higher probability of developing oral cancers regardless of HSCT when compared to those patients that do not require HSCT. Matched mutational studies on FA patients undergoing or not undergoing HSCT are needed to clarify this issue. Thus, FA represents a pathological situation where mutations affecting DNA repair mechanisms stimulate an innate immune/inflammatory response that is critical to disease development, phenotype, and progression.

### 3.2. Complement (A Genetic Alteration of an Immune System Component)

The complement system is one of the oldest innate immune programs maintained throughout evolution. It can be activated by one of three cascading pathways, leading to two major outcomes: (i) the tagging of potential pathogens with C3b, thereby enhancing opsonization by APCs; and (ii) assembly and activation of the membrane attack complex (MAC), which serves to perforate cell membranes of invading pathogens [59]. Mutations or deletions in complement proteins have been associated with a wide variety of diseases. Within families, mutations in complement genes can have diverse outcomes in relation to the individual, but all pathological outcomes are immune/inflammatory-related. Deficiencies in later classical cascade components necessary for the formation of the MAC (C5–C9) are mainly associated with severe recurrent bacterial infections [60,61,62], but, in contrast to complement proteins C5–C8, variants of complement C9 have also been associated with age-related macular degeneration [63]. Unlike late cascade component deficiencies, deficiencies in early complement cascade components C1–C4 are associated with severe immune complex pathologies that bear semblance to systemic lupus erythematosus (SLE), with or without associated glomerulonephritis [64,65,66,67,68,69,70,71]. Deficiencies in these early components impedes phagocytosis by macrophages and neutrophils and the removal of apoptotic debris. Accumulation of these debris is believed to be the main culprit leading to the development of autoantibodies and auto-inflammation that are observed in this pathology. Many self-neoantigens that are not efficiently removed from circulation and the associated auto-inflammation promote tissue destruction, producing additional apoptotic debris, thereby setting up a chronic inflammation cycle [72]. Moreover, similar to C9, diverse variants of C2 and C3 proteins are associated with age-related macular degeneration [63,73]. 

As might be expected, related pathologies are not limited necessarily to defects in the complement proteins themselves, but also arise from mutations in complement regulatory proteins and complement receptors involved in both classical and alternative cascade pathways. Variants of complement factors (B, D, H, and I) are associated with age-related macular degeneration and atypical hemolytic uremic syndrome, while deficiency of these factors leads to recurring bacterial infections [73,74,75,76,77,78,79,80]. Variants of the plasma protease C1 inhibitor result in hereditary angioedema [81,82], while variants of the type 2 C3 receptor, which is present primarily on B-, T-, and various APCs, can either result in SLE or hypogammaglobulinemia as a result of impaired B-cell differentiation [83,84]. Variants of C1q subunit binding protein (C1QBP) result in mitochondrial respiratory chain deficiencies that produce a pathology with multisystemic features [85]. Thus, mutations directly altering innate immune regulatory proteins and the immune/inflammatory response can chronically promote inflammation and/or hinder immune resolution, favoring disease development. Taking into account the nature of mutations in the complement system and the highly complex effects that result, the number of pathologies associated with complement defects (and components of the innate immune system for that matter; see TLR7 [86], PKR [87,88] and PACT [89]) will continually evolve, but to date, other than those listed above, this includes Alzheimer’s, diabetes mellitus, rheumatoid arthritis (RA), systemic scleroderma (SS), and cancer. For a more detailed discussion of the complement systems role in various diseases, see the following Refs. [90,91,92,93,94,95]. 

### 3.3. Environmental/Behavioral Factors

Extrinsic factors also play a significant role in inflammatory-mediated disease. These factors can typically be classified as pathogenic organisms (viruses, bacteria, mold/fungus, etc.), environmental toxins (chemicals, natural sources of radiation, pollution), or behavioral (smoking, alcohol consumption, etc.). 

#### 3.3.1. Pathogenic Organisms

Both human immunodeficiency virus (HIV) and herpes viruses have been associated with neuroinflammation and Alzheimer’s disease, while hepatitis B and C viruses (HBV and HCV) have been associated with hepatocellular carcinoma (HCC) and a number of additional cancers [96,97,98,99]. The stimulation of antiviral/inflammatory pathways, including RIG-I, MDA5, GAS, and PKR, during infection causes the release of cellular factors from damaged cells, along with the ultimate recruitment of inflammatory macrophages, B- and T-lymphocytes. Cytotoxic cytokine synthesis (IFNγ, TNFα, IL-1α/β, IL-6, etc.), in conjunction with viral anti-host mechanisms, often establish cyclical host-viral conditions that are perfect for chronic inflammation. Similarly, bacterial strains such as *Helicobacter pylori* and *Borrelia* sp. are known to be contributing factors to digestive tract cancers and the systemic chronic inflammatory pathology Lyme disease, respectively [100,101]. Inflammation arising from a general bacterial infection (*Staph.* sp., *Strep.* sps.) primarily stems from Toll-like receptor activation, recruited inflammatory APCs, B- and T-cells, secreted cytokines and factors released from damaged and dying cells, while cytosolic PRRs take a more secondary signaling role downstream of TLR activation. In contrast, cytosolic PRRs play a more significant role in the response to intracellular bacterial infection or following phagocytic ingestion of bacterial pathogens [102]. 

More recently, microbiome research has begun to highlight the role that resident bacteria play in controlling gut inflammation [103]. Fecal transplantation is now being critically evaluated as an effective treatment to ulcerative colitis, a chronic inflammatory disorder, which can have both bacterial and viral origins [104]. Of extreme interest are recent studies demonstrating the presence of an altered microbiome at tumor sites that actively contribute to the inflammatory tumor microenvironment [105]. Other than acting solely as a pathogenic agent, it has been speculated that the major effects mediated by the microbiome result from metabolites produced by the resident organisms. Some of these metabolites have been demonstrated to produce changes in the gut mucosa, aiding in the establishment of an inflammatory niche that is favorable for tumor growth [103,105]. Of course, pathological effects due to the microbiome are considered the result of dysbiosis; the symbiotic relationship is discussed in Section 4.

#### 3.3.2. Environmental Toxins

Toxin exposure (asbestos or polychlorinated biphenyls (PCBs)) has long been known to be related to disease. Asbestos toxicity was first noticed in the early 1900s. Since then, asbestos toxicity has been associated with lung inflammation and scarring, lung cancer, pharyngeal cancer, cancer of the larynx, and mesothelioma (a rare aggressive cancer) [106,107]. Asbestos toxicity is also linked to ovarian cancer and gastrointestinal manifestations [108]. Diverse hypotheses to explain how asbestos causes disease each point to the ability of asbestos fibers to stimulate an immunological response that involves elevated production of reactive oxygen/nitrogen species (ROS/RNS) by phagocytic cells (macrophages, neutrophils, and NK cells) and associated DNA damage [106,109].

The major mode of action of toxins, such as PCBs, is through the activation of the aryl hydrocarbon receptor (AHR), a cytosolic transcription factor that is normally inactive until binding of a substrate. Upon substrate binding, AHR is translocated to the nucleus where it stimulates gene expression by both NF-κB-dependent (RELB) and -independent means. Many of the genes regulated by AHR are genes involved in metabolism and elimination of diverse chemical toxins, as well as mitochondrial respiration, which often have significant effects on ROS production [110]. In addition, to the direct metabolic effects, AHR was also demonstrated to regulate the expression of innate immune genes, including IL-1β [111,112].

#### 3.3.3. Behavioral Factors

Alcohol consumption (mainly wine) has had a mixed history being promoted for some health benefits, with moderate consumption, while at the same time being associated with liver disease and several cancers [113]. Higher levels of the detoxifying enzyme alcohol dehydrogenase (ADH) were, until recently, believed to be more protective against the negative effects of alcohol, but recent studies have demonstrated that the reaction catalyzed by ADH, the conversion of ethyl alcohol into acetaldehyde, produces a more toxic compound, which, beyond the ability to cause general cell damage, has significant DNA damaging properties as well [114,115]. Moreover, this effect can be exacerbated by mutations affecting aldehyde dehydrogenase 2 (ALDH2) and the composition of the oral-gastrointestinal microbiota [116]. The induced DNA/cellular damage following alcohol consumption results in activation of DAMPs and PAMPS and an inflammatory response, thus linking chronic alcohol consumption to chronic inflammatory signaling.

Tobacco smoke contains an estimated 5000 chemical compounds, many of which are toxic to cells [117]. Chronic exposure to these compounds either directly or via second-hand smoke has been shown to have immunomodulatory activity, with some compounds enhancing inflammation and others suppressing immunity [118,119]. In general, smoking tobacco induces the recruitment and activation of inflammatory macrophages and natural killer (NK) cells to the lung, producing a pro-inflammatory environment [118]. Interestingly, in chronic smokers an immunosuppressive state is often established whereby the reactivity of antigen-presenting cells is suppressed [120]. This tends to follow a general trend in the development of most cancers where a pro-inflammatory, pro-apoptotic environment is replaced by immunosuppression, in many cases through the presence of suppressive T-cells (Tregs). Interestingly, while this paradox is often associated with cancer, the lack of immunosuppression is associated with chronic auto-inflammatory/degenerative diseases, such as chronic obstructive pulmonary disease (COPD) [121,122].

As previously stated, the effects contributed by environmental and behavioral factors throughout life along with less efficient cell/tissue repair mechanisms are driving forces in age-related pathologies. Moreover, much of the pathological outcome due to environmental factors can also be dictated by predisposing genetic factors at birth as well as any alterations accumulated during one’s lifespan (ex. mutations in ALDH2 and alcohol consumption significantly enhance cancer risk). 

## 4. Adaption vs. Disease in Chronic Inflammation

Is inflammation simply a response to disease and damage or does it have a more vital role in the preservation of an organism as well as the species? Can chronic stimulation of the innate immune/inflammatory response favor adaptation to a changing environment in the best of cases or result in (or promote) disease in the worst of cases? A hallmark of species evolution, as put forth by Jean-Baptist Lamarck and Charles Darwin, is the adaption of species to their environment through natural selection. At the heart of this process is the acquisition of an advantageous characteristic through non-random (Lamarck) or random (Darwin) genetic change that makes one or more members of a species better suited to a given environment, eventually leading to a divergence of species [123]. Based on the recent evidence from studies of rapid climate change, this process may neither be totally random (passive) nor non-random (active). Both the rate at which certain species are acquiring mutations, as well as the preferential sites of many mutations, suggests that the process of natural selection and evolution may actually be, in part, an active process [124,125]. Certain behaviors and environmental changes have been shown to affect the epigenetic programming of gametes, as well as that of the whole organism, but what is the over-riding regulator of this process [126,127]?

The interaction of organisms at almost every level with their surrounding environment is tutored by some form of immune response. Infectious agents, toxins, and other environmental stresses induce an innate immune inflammatory response, which in the acute phase has the objective of protecting the organism from the stressing agent by removing the stressor and repairing any damage, as stated above. These are transient stresses that the organism either is able to overcome and re-establish homeostasis or succumbs within a relatively short period of time. However, what happens when the stress is sub-lethal and yet cannot be resolved? As prolonged inflammation/inflammatory signaling generally has a negative impact on cells and tissues, chronic activation of the associated pathways can ultimately lead to chronic damage and cell death, with continual homeostatic imbalance and even death of the organism/species (in extreme cases depending on the stress). Thus, chronic inflammation can establish a selective pressure unless some means of adapting to the given stress can be achieved. Studies on a smaller scale have shown a similar scenario plays out in the cancer micro-environment, with the development or emergence of cancer stem cells [128]. The unrelated, random acquisition of a mutation at this point could be beneficial in establishing an adaptation to the stress, harmful by promoting disease, both, or neither. Most strict adaptations or silent modifications are not observed unless by chance, as healthy individuals are not often biomedical study subjects, and genetic longitudinal studies over a lifetime to catch these alterations are not practical. In contrast, there are multiple examples where mutations are associated with adaptation and disease or disease alone. 

### 4.1. Bone Marrow Failure Disorders and Acute Myelogenous Leukemia

One of the more interesting adaptation/disease models is that provided by bone marrow failure disorders. The initial underlying etiology of these disorders is diverse, ranging from gene mutations (FA, myelodysplastic syndromes (MDS), Diamond-Blackfan Anemia (DBA), Shwachman-Diamond Syndrome (SDS), aplastic anemia (AA)), environmental effectors (AA), and unknown factors (MDS, AA) [12,129,130,131]. Age and prior therapeutic treatment with antineoplastics also plays an important role in MDS. In the early phase, there is an establishment of a pro-inflammatory/pro-apoptotic bone marrow environment, which initially leads to the cell death of hematopoietic progenitors and the associated anemia. In severe cases of anemia, affected individuals become dependent on transfusions for survival. At some point during disease progression, progenitor cells lose sensitivity to the pro-inflammatory/apoptotic environment and begin to expand to the peripheral blood, eventually culminating in AML. During this transition, adaptive changes/mutations occur to promote the resistance of hematopoietic progenitor cells to the pro-inflammatory environment [12,130,131,132]. This loss of apoptotic control happens to be one of the main initiating events in the formation of tumors, and under conditions of inflammation can result in a population of cells which no longer have the ability to respond to the growth control branch of inflammatory signaling while still maintaining the ability to respond to tissue repair, survival, and proliferative signals. 

Recent data demonstrated that innate immune/inflammatory signaling has a strong influence on AML progression. Elevated levels of active PKR in bone marrow derived progenitor cells from FA and MDS patients were first reported in 2004 and 2008, respectively [45,133]. In MDS, the number of bone marrow derived mononucleated cells (BMMCs) that contained elevated levels of active PKR were found to increase with increasing International Prognostic Scoring System (IPSS) risk categorization of the patient from which the BMMCs were derived. Not only was more active PKR present, but active PKR was localized primarily to the nucleus in BMMCs from high-risk patients [133]. Similarly, active PKR was observed in both the nucleus and cytosol in acute leukemic cells where PKR activity was determined to be necessary for leukemic cell growth [134,135]. These initial studies suggested that activated innate immune/inflammatory signaling could have a promoting effect on tumor development, but how this might occur was not clear until 2015. PKR, whose expression and/or activation is elevated in diverse tumors, was demonstrated to accelerate leukemic development [8,12,136]. In AML patients, PKR expression was directly correlated with poor overall survival and shorter time of remission, regardless of the presence of unfavorable cytogenetics. The authors determined that PKR activation enhanced the rate of mutagenesis by suppressing ataxia-telangiectasia mutated (ATM) kinase, an enzyme key to DNA double-strand break repair, thus promoting MDS progression to AML [136] (Figure 1b). Thus, activation of PKR could favor an enhanced rate of mutagenesis. Unfortunately, in this case, PKR activation and enhanced mutagenesis promoted continuous clonal survival and expansion, resulting in disease progression and death of the organism.

### 4.2. Hemoglobin Beta

Of course, there are also cases where the opposite is held true, where a random mutation’s emergence in a population is promoted by an environmental factor. Among one of the most prominent adaptation/disease mutations in this category is that involving hemoglobin beta (HBB) mutations associated with sickle cell anemia (HbS), in global regions prone to malaria. In these areas, the sickle cell mutation has been selected as an adaptation to an infective pathogen. While these mutations offer a level of resistance to malarial infections and disease, the associated phenotype can be deadly in individuals homozygous for HBB mutation. The general theory holds that the acquisition of HBB mutations occurred by random “happenchance” that, in regions with high incidence of malaria, became a favorable trait. In regions where malaria was not present, similar mutations would have been eventually deleted from the population pool. However, is it possible that the high incidence of malarial infection in these regions over the long-term may have favored the original emergence of these mutations in some manner? In a direct manner, no, but a recent study has shown that the acquisition of the HbS mutation in Africans is not totally random [137]. In the associated study, the authors reported that the mutations at this locus in Ghanaians occurred at a higher frequency than that observed in Europeans, suggesting that long-term environmental selective pressure exerted by the presence of malaria has favored mutagenesis at this locus. 

### 4.3. Gastro-Intestinal Microbiome

The importance of the relationship between the microbiome composition and host health has increasingly become more apparent. Individuals begin to acquire microbial species at birth, starting with maternal-associated oral–fecal/vaginal and later breast-feeding routes. Later in life, diet, behavioral activities, and lifestyle have major influences on the bacterial species represented in the microbiome. The interaction between the host immune system and the microbes present can establish a “balance of power” whereby immune inflammatory responses are controlled as well as the composition and expansion of microbial species present, creating a microenvironment beneficial to both host and microbe. Two of the most important routes of host control over the microbiome are the production of pancreatic-derived antimicrobial peptides (AMPs) and the synthesis of secretory IgA [138]. In contrast, the surface proteins and carbohydrates of the bacterial species present stimulate a specific set of Toll-like receptors, while other TLRs are completely absent, down-regulated, or transient in expression. Activation of the TLRs in the context of a healthy microbiome stimulates the concomitant activation of the phosphatidylinositol-3 kinase-AKT pathway, promoting the downstream synthesis of anti-inflammatory mediators. In addition, bacterial species composing a healthy microbiome appear to be adept at stimulating regulatory T lymphocytes, thus promoting immune suppression. Alterations in either of these host-mediated regulatory mechanisms can promote the appearance of a pro-inflammatory microbiome [138].

While healthy eating habits and lifestyle have a significant symbiotic effect on the bacterial species present, and these species tend to promote an anti-inflammatory environment, repressing innate immune inflammation and maintaining the health of the gut epithelium, poor eating habits and lifestyle, as well as antibiotic usage and physical and/or mental stress, alter the microbiome in a negative way. This change in the microbe composition likely relates to both changes in the host relation to the microbiome (i.e., AMPs or other host metabolites synthesized) and the capacity of certain microbial species to better flourish under the altered conditions. As stated in Section 3.3.1, the species present and the metabolites produced by this altered microbiome can often promote local inflammation, eliciting a stress response, chronic tissue damage, and disease. In addition to the gut microbiome, commensal microbes have also been observed to infiltrate and colonize other tissues of the gastrointestinal tract, such as the bile ducts, gall bladder, and liver, and to be important for the health of these tissues [139,140]. 

### 4.4. Innate Immune Control of DNA Damage Repair and Epigentic Modifications

Current data from disease models are beginning to suggest that chronic stresses can actively favor mutagenesis and that the mutations that appear are not such a random happenchance after all, as they appear in particular regions of the genome at rates higher than would be predicted by random happenchance [124]. Thus, if chronic stress leads to chronic inflammation, what better way to control the rate of mutagenesis to favor adaptation than through components of the innate immune system? 

Other than PKR, a number of additional innate immune/inflammatory and interferon regulated factors have roles in both DNA damage repair and epigenetic modification, including ADAR1, the IFNγ-inducible protein 16 (IFI16), and the three prime repair exonuclease 1 (TREX1) [55,141,142,143,144] (Figure 1b). Moreover, a study by Tassinari et al. demonstrated that N^6^-adenosine methyltransferase METTL3, an epigenetic modifier shown to have a significant role in the DNA damage response and gametogenesis, methylates ADAR1 mRNA, enhancing ADAR1 protein synthesis [145,146,147]. The link between innate immune/inflammatory stress and DNA repair/modification may suggest that under conditions of constitutive/chronic stress, the immune response opens the door to a potential escape mechanism, allowing for enhanced mutation rates in the off-chance that an adaptation to the stress may be achieved [55,56,136,143]. In an individual organism this may occur throughout one’s life, but how could such a mechanism favor transmission to subsequent generations? The effect of stress on offspring has long been reported in models such as fruitflies, *C.elegans*, and even birds [148,149,150], but recent data suggest that stress and inflammation also influence the methylation and epigenetic modification of gamete DNA, especially that of sperm [126,127,151]. In addition, stress and inflammation are bound to affect the mother and the environment of the developing fetus.

So, how could such selective mutagenesis result in degenerative diseases? In general, progression of degenerative disease is not measured by clonal evolution of cells in the affected tissue but by the degree of tissue destruction and patient scores from performance tests. This is not to say that clonal selection does not take place, it does, but almost strictly in cells belonging to the adaptive immune response (T and B cells) [152]. In pathological tissue specimens from patients with various degenerative diseases, the T cell population is pro-inflammatory. Moreover, in some cases there is a tendency for the appearance of B cells producing disease specific autoantibodies [153,154]. As regards the innate immune system, autoinflammatory and degenerative diseases tend to have an altered type I interferon response (IFR) or alterations in proteins belonging to the IFR [12,154,155,156]. Finally, an accumulation of data from SARS-CoV2 studies, as well as studies into AD, PD, and idopathic myocitis, suggest a role of altered PI3K/AKT/GSK3 signaling affecting the innate immune/interferon response in these pathologies [157].

## 5. Conclusions

The relationship between inflammation and disease should no longer be considered linear with disease leading to chronic inflammation, but the chronic stress-inflammation cycle itself should be viewed as a potent contributing factor to disease inception and progression. Indeed, disease may be the result of a failed attempt (or an accumulation of failed attempts) to adapt to a given stress (or stresses), a probability bound to increase with the number of stresses encounter during one’s lifespan and compounded by defects in a continually aging system. A clearer understanding of innate immune signaling and its relation to epigenetic modifications, DNA damage repair pathways, and their influence on adaptive immunity is likely to open the door to new therapeutic possibilities that will allow us to treat cancer without encountering drug-resistance or re-establish tissue homeostasis in auto-inflammatory/degenerative diseases.

## Data Availability

Not applicable.
